# Infective Endocarditis Caused by Serratia marcescens: A Case Report and Literature Review

**DOI:** 10.7759/cureus.96182

**Published:** 2025-11-05

**Authors:** Mohammadshah I Gul, Bara M AL-Qudah, Latifa AlMahmoud, Monder M Karayem, Alhady A Yusof, Abdulqadir J Nashwan

**Affiliations:** 1 Internal Medicine Department, Hamad General Hospital, Doha, QAT; 2 Medical Intensive Care Unit and Emergency Department, Hamad Medical Corporation, Doha, QAT; 3 College of Medicine, Weill Cornell Medicine Qatar, Doha, QAT; 4 Nursing and Midwifery Research Department, Hamad Medical Corporation, Doha, QAT

**Keywords:** cardiothoracic infections, endocarditis management, infective endocarditis, mitral valve replacement, sepsis, serratia marcescens

## Abstract

*Serratia (S.) marcescens* is an opportunistic, gram-negative pathogen most commonly associated with respiratory tract, urinary tract, and soft-tissue infections. Reports of *S. marcescens* as a causative agent of infective endocarditis are rare, especially when compared with the more frequently implicated pathogens such as *Staphylococcus* and *Streptococcus* species. The clinical presentation, diagnostic challenges, and therapeutic considerations of *S. marcescens* endocarditis remain poorly characterized in the literature. This case report aims to provide a detailed description of an unusual presentation of *S. marcescens* infective endocarditis and to review previously reported cases to highlight common features, treatment strategies, and outcomes.

We report the case of a middle-aged male with a significant past medical history of atrial fibrillation managed with warfarin and mitral valve replacement for rheumatic mitral stenosis. The patient was initially admitted under the neurosurgery service with bilateral subdural hematomas and underwent a right-sided mini-craniotomy with evacuation and subdural drain insertion. Three weeks postoperatively, he developed hypotension and fever. Blood cultures grew *S. marcescens*, and transesophageal echocardiography (TEE) revealed a 7 × 3 mm vegetation on the prosthetic mitral valve. The patient was treated with a combination of intravenous meropenem and gentamicin for a total of four weeks. Follow-up TEE demonstrated complete resolution of the vegetation, and the patient had an uneventful recovery.

## Introduction

*Serratia marcescens* is an opportunistic gram-negative pathogen that frequently causes respiratory tract, urinary tract, and soft-tissue infections, particularly in immunocompromised patients [1]. Its virulence is mediated by fimbriae-like adhesins that facilitate surface attachment and biofilm formation, enhancing persistence in host tissues [2]. Compared with typical pathogens associated with infective endocarditis (IE), such as *Staphylococcus *and *Streptococcus *species, *S. marcescens* has been associated with higher mortality rates [1,3]. Reports of *S. marcescens* as an etiological agent of IE remain limited, which can delay recognition and treatment. Additionally, its ability to produce beta-lactamase and extended-spectrum beta-lactamases (ESBLs) contributes to multidrug resistance, complicating antibiotic selection and clinical management [4]. Early recognition and timely intervention are therefore essential to improve outcomes.

## Case presentation

A 56-year-old Sri Lankan man with a history of rheumatic heart disease status post bi-leaflet mechanical mitral valve replacement six months prior, permanent atrial fibrillation on anticoagulation, type 2 diabetes mellitus, and prior cerebrovascular accident presented with a one-week history of severe headache. Computed tomography (CT) of the head revealed bilateral subdural hematomas, and the patient underwent evacuation with subdural drain insertion.

During his hospital stay, he developed fever, hypotension, and tachycardia unresponsive to fluid resuscitation, prompting transfer to the medical intensive care unit (MICU). Vasopressor support with phenylephrine was initiated. On examination, the patient was alert and oriented to time, place, and person. Pupils were equal and reactive to light. Chest auscultation revealed equal bilateral air entry, without crepitus, rhonchi, or added heart sounds; S1 and S2 were normal, with no audible murmurs or pericardial rub. Abdominal examination was unremarkable, with a soft, non-tender abdomen, active bowel sounds, and no organomegaly. The initial laboratory findings revealed leukocytosis, anemia, hyponatremia, hypoalbuminemia, and elevated inflammatory markers (Table 1).

**Table 1:** Laboratory results on admission WBC, white blood cell count; RBC, red blood cell count; Hgb, hemoglobin; Hct, hematocrit; MCV, mean corpuscular volume; Plt, platelet count; ANC, absolute neutrophil count; Cr, creatinine; Na, sodium; K, potassium; Cl, chloride; HCO₃, bicarbonate; Ca, calcium; T. Bil, total bilirubin; TP, total protein; Alb, albumin; ALP, alkaline phosphatase; ALT, alanine aminotransferase; AST, aspartate aminotransferase; Chol, cholesterol; TG, triglycerides; Trop-T HS, troponin-T high sensitivity; CRP, C-reactive protein; PT, prothrombin time; INR, international normalized ratio; APTT, activated partial thromboplastin time.

Parameter	Value	Reference Range (units)
Hematology		
WBC	12.5	4.0–10.0 × 10³/µL
RBC	3.5	4.5–5.5 × 10⁶/µL
Hgb	10.6	13.0–17.0 g/dL
Hct	31.6	40.0–50.0 %
MCV	90.3	83.0–101.0 fL
Plt	217	150–410 × 10³/µL
ANC	11.4	2.0–7.0 × 10³/µL
Biochemistry		
Urea	8.2	2.5–7.8 mmol/L
Cr	97	62–106 µmol/L
Na	127	133–146 mmol/L
K	4.2	3.5–5.3 mmol/L
Cl	94	95–109 mmol/L
HCO₃	20	22–29 mmol/L
Ca	2.03	–
T. Bil	28	0–21 µmol/L
TP	57	60–80 g/L
Alb	26	35–50 g/L
ALP	72	40–129 U/L
ALT	18	0–41 U/L
AST	13	0–40 U/L
Chol	6.3	<5.2 mmol/L
TG	2.2	<1.7 mmol/L
Trop-T HS	21–12	3–15 ng/L
CRP	161.8	0–5 mg/L
Coagulation Profile		
PT	16.9	9.4–12.5 sec
INR	1.4	~1.0
APTT	142	25.1–36.5 sec

**Figure 1:**
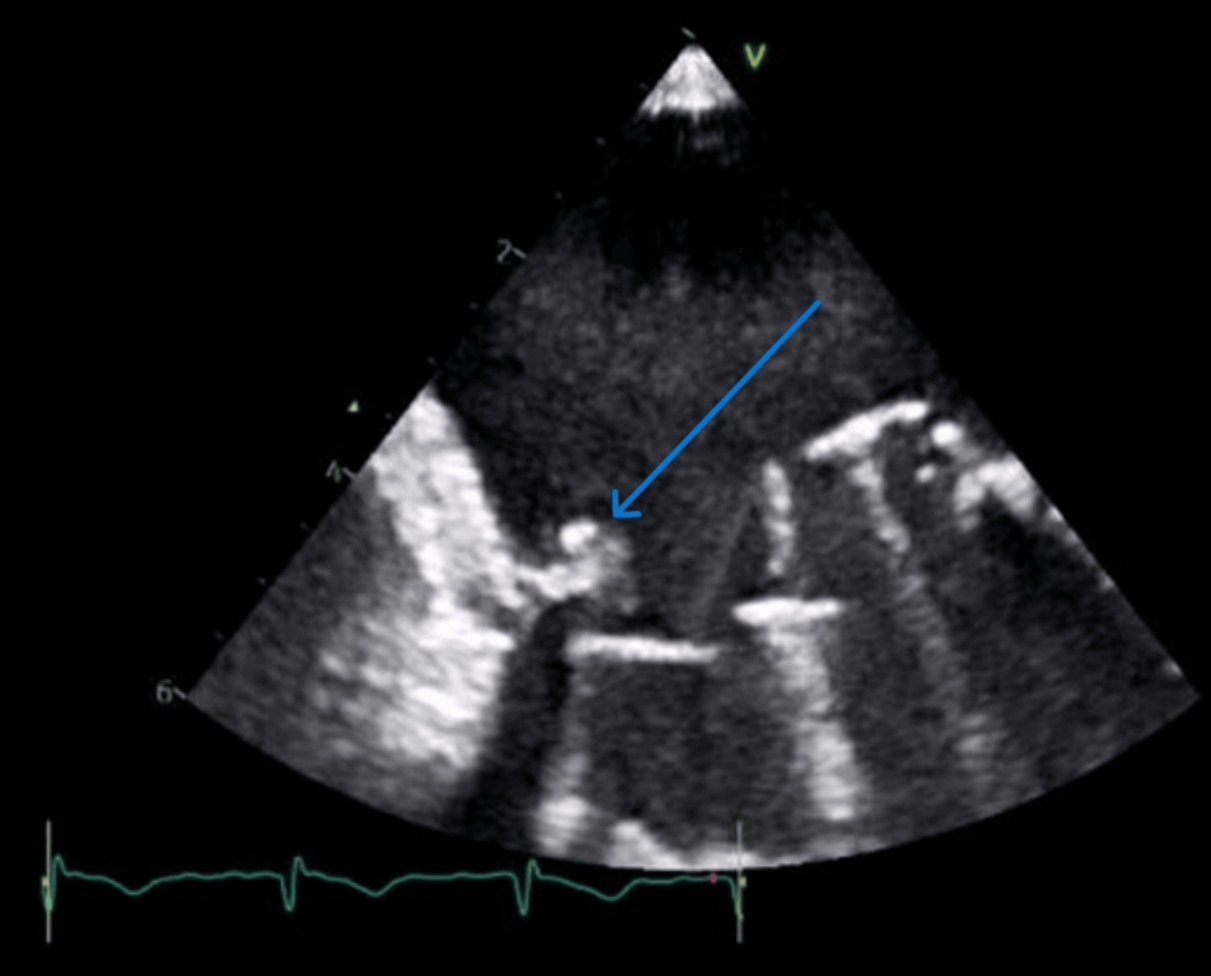
TEE showing 7x3mm vegetation attached to the metallic mitral leaflet TEE: transesophageal echocardiogram

A sepsis workup was initiated, and empiric intravenous meropenem and vancomycin were started. Blood cultures subsequently grew *Serratia marcescens*. Transthoracic echocardiography (TTE) revealed no vegetations; however, given the history of prosthetic mitral valve replacement, transesophageal echocardiography (TEE) was performed, demonstrating a 7 × 3 mm vegetation attached to the metallic mitral leaflet (Figure 1 and Video 1).

**Video 1:** Transesophageal echocardiography (TEE) findings Initial TEE: Demonstrated a 7 × 3 mm mobile vegetation attached to the metallic mitral valve leaflet, consistent with prosthetic valve endocarditis. Follow-up TEE (post-treatment): Showed complete resolution of the vegetation with no residual mass or regurgitation, confirming successful treatment.

The infectious disease team was consulted, and they advised continuing meropenem (1000 mg, IV infusion, every 8 hours), adding gentamicin (started with 80 mg IV infusion TID, then increased to 100 mg IV infusion every 8 hours based on gentamicin levels), and stopping vancomycin. The cardiothoracic team advised no surgical intervention, continuation of antibiotics, and a follow-up TEE. Repeat blood cultures obtained after 48 hours were negative. On day seven, the patient experienced a single febrile episode; repeat blood cultures were positive for *Actinobacteria*. Ciprofloxacin was added to the existing regimen of meropenem and gentamicin. Contrast-enhanced CT abdomen showed no intra-abdominal collection or pelvic fluid. A follow-up TEE performed three weeks later demonstrated complete resolution of the vegetation. The infectious disease team recommended completing a total of four weeks of intravenous antibiotics, after which the patient remained clinically stable. The patient was frequently seen in the cardiology clinic. After a visit one year later, he was healthy, with no recurrent attacks of fever or infective endocarditis, and doing well.

## Discussion

*S. marcescens* is an uncommon but clinically significant cause of infective endocarditis, particularly in patients with prosthetic heart valves or pre-existing cardiac disease [1,5]. Its opportunistic nature, combined with intrinsic and acquired resistance mechanisms, creates significant therapeutic challenges. Although IE due to *S. marcescens* remains rare compared with *Staphylococcus aureus* or *Streptococcus species*, its association with hospital-acquired infections and invasive procedures highlights its importance as a healthcare-associated pathogen [6].

This organism belongs to the *Enterobacteriaceae *family and exhibits several virulence factors, including biofilm formation and production of hydrolytic enzymes such as beta-lactamase, lipases, and proteases [2,4,7]. Biofilm development on prosthetic material is central to its pathogenesis, enabling persistent colonization and resistance to both host immune defenses and antimicrobial therapy [7]. In our patient, the presence of a prosthetic mitral valve likely provided a substrate for colonization and biofilm formation, leading to the development of vegetation despite broad-spectrum empiric therapy.

Management of *S. marcescens* endocarditis is complicated by its resistance profile. The production of extended-spectrum beta-lactamase (ESBL) limits the utility of penicillins and cephalosporins, making carbapenems and aminoglycosides preferred therapeutic options [4,8]. In our case, combination therapy with meropenem and gentamicin led to clinical improvement and clearance of bacteremia. Ciprofloxacin was added when subsequent cultures revealed additional gram-positive growth, demonstrating the need for the dynamic adjustment of therapy based on culture results.

A review of the literature indicates that most cases of *S. marcescens* endocarditis occur in immunocompromised patients or those with prosthetic valves [1,6,9]. Early surgical intervention is sometimes required, particularly when large vegetations, persistent bacteremia, or embolic complications are present [3]. However, in our case, the vegetation resolved with medical management alone, suggesting that surgery can be avoided in carefully selected patients who respond promptly to antimicrobial therapy.

This case underscores the importance of maintaining a high index of suspicion for atypical organisms when evaluating patients with prosthetic valves and systemic infection. Early echocardiography, repeated blood cultures, and multidisciplinary involvement (infectious disease, cardiology, and cardiac surgery) are essential to achieving favorable outcomes [8,10]. Continued research is needed to establish standardized treatment protocols and clarify the role of surgery versus prolonged medical management in *S. marcescens* endocarditis.

## Conclusions

In conclusion, *S. marcescens* endocarditis, although rare, represents a challenging clinical entity due to its association with healthcare settings, ability to form biofilms on prosthetic material, and multidrug resistance. This case emphasizes the need for vigilance in identifying atypical pathogens in endocarditis and adapting treatment strategies accordingly. A collaborative approach involving microbiology, infectious disease, cardiology, and potentially surgical teams is essential to optimize outcomes for patients affected by this rare but serious infection.
